# Reflexive standardization and the resolution of uncertainty in the genomics clinic

**DOI:** 10.1177/03063127231154863

**Published:** 2023-03-15

**Authors:** Adam Hedgecoe, Kathleen Job, Angus Clarke

**Affiliations:** Cardiff University, Cardiff, UK

**Keywords:** genomics, whole genome sequencing, whole exome sequencing, variants of uncertain significance

## Abstract

In genomics, the clinical application of Next Generation Sequencing technologies (such as Whole Genome or Exome Sequencing) has attracted considerable attention from UK policymakers, interested in the benefits such technologies could bring the National Health Service. However, this boosterism plays little attention to the challenges raised by a kind of result known as a Variant of Uncertain Significance, or VUS, which require clinical geneticists and related colleagues to classify ambiguous genomic variants as ‘benign’ or ‘pathogenic’. With a rigorous analysis based on data gathered at 290 clinical meetings over a two-year period, this paper presents the first ethnographic account of decision-making around NGS technology in a NHS clinical genomics service, broadening our understanding of the role formal criteria play in the classification of VUS. Drawing on Stefan Timmermans’ concept of ‘reflexive standardisation’ to explore the way in which clinical genetics staff classify such variants this paper explores the application of a set of criteria drafted by the American College of Medical Genetics and Genomics, highlighting the flexible way in which various resources – variant databases, computer programmes, the research literature – are drawn on to reach a decision. A crucial insight is how professionals’ perception of, and trust in, the clinical practice at other genomics centres in the NHS, shapes their own application of criteria and the classification of a VUS as either benign or pathogenic.

## Backing genomics to the hilt

Since then Prime Minister David Cameron’s launch of a scheme to sequence 100,000 genomes in 2012 (Prime Minister’s Office, 2012), the profile of next generation sequencing technologies in NHS clinical settings has steadily grown. The ‘100,000 genome project’ was explicitly framed as the start of significant structural changes within the NHS, more recent indicators of which might include reshaping the pre-existing regional genetics centres into a more centrally coordinated ‘National genomics medicine service’ and the concentration of the Chief Medical Officer’s 2016 annual report on the potential of genomics in the health service (Davies, 2017; NHS England, 2017). Summarizing this genomic enthusiasm, the Secretary of State for Health and Social Care boasted in an April 2021 speech at the Association of the British Pharmaceutical Industry (ABPI) annual conference: ‘We’re making genomic sequencing a routine part of everyday diagnosis and treatment, and the UK again is uniquely placed. The NHS has the scale and the systems to make it happen … we’ll be backing genomics to the hilt’ (Hancock, 2021).

The umbrella term ‘next generation sequencing’ covers a number of different ways of looking at a person’s genome – the complete set of genetic information contained in their chromosomes – which tend to share parallel investigations of large amounts of genomic materials and which can be best distinguished in terms of the scale at which they work. The narrowest approach is offered by ‘gene panels’ which involve the sequencing of a pre-determined set of genes, usually grouped together around specific conditions (e.g., a ‘cardiomyopathy panel’). Broadening outwards is whole exome sequencing (WES), which focuses on the 1–2% of the genome that codes for protein (the exome) and which is estimated to contain around 85% of disease-causing mutations. This is seen as a cheaper – but also more clinically relevant – alternative to whole-genome sequencing (WGS) (Seaby et al., 2016). Finally, in addition to these approaches, we might add more structural assessments of the genome, such as array comparative genomic hybridization (Array CGH) which, while not employing sequencing, do provide a genome-wide review of the number of copies of chromosomal elements.

Alongside the question of what is sequenced, there is also the question of what portion of the sequence generated is to be analysed and interpreted. WES or WGS may be used as a platform for the operation of virtual gene panels, where only the specific, pre-selected sequence elements are interpreted despite the whole exome or genome sequence having been generated. The first steps of interpretation are processed by IT systems; those deviations from the usual sequence that are identified by algorithms as potentially pathogenic are then flagged up to be interpreted in greater depth by human operators (bioinformaticians, laboratory scientists or clinicians). Much of the current clinical application of WGS in fact entails the use of large gene panels with no attempt being made to interpret all sequence variants, which remain in patient records or research databases for potential future analysis. This has many benefits for the clinical service: Time and attention is spared from having to focus on uninterpretable variants but, when ‘new’ genes are implicated in a disorder, it is possible to conduct a reanalysis that incorporates this updated information.

## VUS, Uncertainty and the nature of genomic sequencing

While there is considerable excitement about these technologies at the policy level, clinical professionals are more cautious (Feero 2014; for a detailed analysis see Kerr et al., 2019). A crucial component of this professionalized caution comes from experience with an everyday aspect of next generation sequence technologies – though one largely overlooked in more ebulliant policy accounts (e.g. Davies, 2017) – the steady accumulation of results known as VUS, or variants of uncertain (or unknown) significance. These are identified changes (variants) in a specific gene (in comparison to a reference genome), where the impact on its carrier is not known.

Initially identified by geneticists and oncologists using BRCA 1 and 2 testing in breast cancer, with the ‘ever-growing accumulation of genetic data generat[ing] larger and larger percentages of VUS’ (Federici & Soddu, 2020, p. 2), such changes have come to be seen as ‘flies in the ointment’ of next generation sequencing (Domchek & Weber, 2008) making ‘clinical management recommendations more complex, while also potentially creating anxiety or misunderstanding among patients’ (Cheon et al., 2014). Given that such a result will be given in a clinical context (e.g. a test of tumour material, or of a child with learning difficulties associated with a genetic syndrome) a number of questions then arise for the commissioning clinician and the lab about such results: ‘Should they be disclosed to patients, and how should the patients be counseled? Should they inform clinical management? What follow-up studies should be done?’ (Hoffman-Andrews, 2017, p. 650). Since VUS are not fixed over time and future information may lead them to be judged as either harmless or pathogenic, there is the added complexity of a ‘moving target’.

Building on the considerable social scientific scholarship exploring the impact of single-gene testing on patients, parents and families, work explicitly addressing decision making around VUS shows how, in resolving these results, groups of professionals – such as Clinical Exome Sequencing (CES) data boards – can be seen as ‘genomic causality brokers’ (Timmermans et al., 2017, p. 450), choosing to ‘report out’ (to the commissioning clinician) a variant as a VUS. In line with other recent work in this area (Kuiper et al., 2022), and contra typical social science accounts of medical uncertainty, these authors represent VUS as useful resources for the clinical collective, as a way of ‘spread[ing] the genetic agenda, creat[ing] new genetic knowledge and keep[ing] patients under genetic purview’ (Timmermans et al., 2017, p. 441).

While this work provides a crucial first step in our understanding of how professionals resolve the challenges around VUS (in particular) and genomic sequencing more broadly, there are significant differences that limit the applicability of these conclusions to the role of genomics in the NHS. For example, the clinical exome service examined by Timmermans and colleagues, while based in an academic centre, was set up in explicit commercial competition with industry providers, shaping infrastructural issues (such as the inclusion of questions about miscarriage in paperwork with an explicit view to IVF couples being an important potential customer base for the service) and decision-making processes around VUS (concerning the use of genomic databases to deal with commercially relevant time constraints) (Timmermans, 2015). There are also differences in terms of the clinical make up (or otherwise) of the exome service (which is predominantly made up of clinical scientists) and NHS genomics meetings (where the majority of participants come from a clinical background and where there is much greater experience of contact with patients), differences which evidence suggests might lead to variation in decision making (Shashi et al., 2016).

## Methods

This article presents data from a Wellcome Trust funded project, ‘Professional decision making around next generation clinical genetics’, which set out to explore how groups of professionals make decisions about uncertain aspects of modern clinical genetics/genomics, and how those uncertainties are communicated to patients. Our approach was ethnographic, sitting in on and audio recording professional meetings across a number of different settings at the same site over roughly a two-year period (September 2017 to November 2019). Meetings varied in terms of frequency (from weekly prenatal to the monthly Genomics MDT meetings), usually lasting between one and two hours. These meetings included: Prenatal testing meetings (n = 87 + 2 ad hoc); Medical Genetics Cases (70); Dysmorphology clinic meetings (37); Developmental delay research meetings (12); Genomics MDT (22); Cancer risk review (25); Cancer Molecular Meeting (10); and Inherited Cardiac disease (25).

The choice of meetings to observe was driven partly by our focus on genomic testing technologies (e.g. Genomics MDT), and partly by an interest in different conditions (i.e. cancer and inherited heart disease).^1^ The nature of the conditions being tested and the organization of the service means that the same case might be presented at a number of these meetings. For example, a test result might enter discussions via a prenatal meeting before being subsequently discussed at both dysmorphology and Genomics MDTs.

The number of professionals attending varied according to the nature of each meeting. Disease specific meetings (inherited heart disease for example) might consist of a couple of cardiac consultants, a consultant clinical geneticist with an interest in heart disease and one or two genetic counsellors. Other meetings – such as the monthly Genomics MDT or medical genetics cases – involved considerably more participants (up to thirty) consisting mainly of clinical geneticists and genetic counsellors, but also some staff from the testing laboratory, all associated with the large local NHS genetics service based in the location we have anonymised as ‘Ernshire’. While attendance varied, membership of the different meetings overlapped considerably, with the same people attending a number of them in any one week.

The format and purpose of these meetings varied according to their function, with some meetings – for example, prenatal – consisting of a brisk overview of the patients currently on that service, with a view to arranging consultation or moving them off the list. Other meetings were more discursive, offering opportunities for senior staff to outline a particularly interesting case, for debates over changes in policy (either at national or local level) or, in the words of Alan, one of the consultant clinical geneticists, to provide a ‘democratic decision to crowd-source ideas’, often around VUS and how to reclassify them.

The meetings we observed were hierarchically ‘flat’ in terms of individuals’ seniority and their right to engage in discussion. While cases were often introduced by more senior colleagues (a consultant clinical geneticist, for example), there was no restriction on more junior staff engaging in discussion. Indeed, given the training role these meetings sometimes played, this was a normal feature of these meetings. Since these junior colleagues were usually seeking advice on how to resolve particular cases, senior clinicians often offered their opinions and shaped the final decisions. However, the need to ‘crowd-source’ interpretations was not restricted to junior clinicians, and it was common for consultant clinical geneticists to seek broader consensus on what to do with a VUS.

Analysis of our data was broadly abductive (Timmermans & Tavory, 2012), and was an extended, iterative process, starting with cases of interest being flagged as such by KJ (who observed the majority of the meetings) at the point of recording. These were then transcribed and re-analysed by AH before further discussion took place with both KJ and AC. The majority of these ‘cases of interest’ were flagged as such because they involved the discussion of a VUS, although other topics (for example broader debates about policy or ethical dilemmas) were also included. Some kinds of meetings – Prenatal or Cancer Risk Review – produced very few of these cases, because of their function and their context within the clinical process. Others, such as the Genomics MDT meetings or Medical Genetics Cases were highly productive, since one of their purposes was to resolve particularly difficult or complex cases (such as VUS). The final paper was drafted by AH with contributions from KJ and AC.

## Reflexive standardization and the impact of the ACMG criteria

A crucial difference between the NHS genomics clinic in our research and previous work on decision-making around VUS is the role played by external standards in decision making. Previous STS scholarship on standards has emphasized ‘how efforts to standardize practice are reinterpreted, accommodated, and resisted at the local level’ with ‘the very procedures intended to quantify and standardize and to make the process more predictable and uniform in fact rendering the local practice even less standard’ (Hogle, 1995, pp. 496, 487).

The clinical exome board members observed by Timmermans (2017) had considerable flexibility in how they approached questions of pathogenicity, uncertainty and what results were reported to patients. Where standards were employed in such decision making, the key insight is that classic depersonalized standardization – as explored by Porter (1996), for example – is not enough in a setting where you cannot rely on clinicians to fill in standardized forms properly, and where the key databases you rely on to filter your gene selections are incomplete and constantly shifting. In this context: ‘the opposition between trust in standards and trust in scientific communities is overly simplistic’. While ‘standards set the parameters of the genotype–phenotype link … they do not determine what will be reported to patients’ (Timmermans, 2015, p. 93). The solution is ‘reflexive standardization’, where direct trust in standards is replaced by trust in specific experts' appropriate use of standards (p. 94). Where such trust is not possible, the scientists come up with practical workarounds: For example, this team ‘suspects that an autism diagnosis is often given incorrectly when developmental delay may be more appropriate. … Any patient labeled autistic is therefore automatically checked for developmental delay, even if the clinician leaves that latter box blank’ (p. 87).

The most important difference between this work and decision making around variants in the NHS is that, unlike Timmerman’s CES professionals, clinical geneticists, counsellors and laboratory staff in the NHS *do* employ a set of standardized criteria to decide which results to report back to clinicians as clinically relevant. This set of criteria, typically referred to as the ‘ACMG criteria’ or ‘ACMG table’ – actually a set of guidelines agreed to by both the American College of Medical Genetics and Genomics *and* the Association for Molecular Pathology (Richards et al., 2015) – has become the required standard for decision making around VUS in NHS settings, largely as a solution to perceived variation in practice between different sites (Raza et al., 2017), with professional bodies in the UK issuing guidance as to how the ACMG criteria can be operationalized (Ellard et al., 2017, 2020).^2^

This article thus provides insight into professional decision making and the challenges to reflexive standardization in the context of the application of system-wide standards, in this case around decision making around VUS within the context of the ACMG criteria.

## Results: How are the criteria applied?

A typical example of a smooth application of the criteria can be found in one Genomics MDT meeting where Alan, a consultant clinical geneticist, sets out the patient’s clinical phenotype:

He has developmental delay, [unclear] squint and he's dysmorphic too and he's got a maternal history of disabilities and he's actually cared for by his father. One physical feature to note is he’s got quite small distal phalange in his hands and feet which give him quite small nails and tapering fingers.^3^

The criteria that the clinical team can apply in this case are set out in Table 1 (Richards et al., 2015). The left-hand column sets out the different kinds of data that can be drawn on to resolve a VUS as either pathogenic or benign, for example, according to one of the various algorithms available (‘computational and predictive data’) or whether the variant in question is seen elsewhere in the patient’s family (‘segregation data’). Different kinds of data contribute different levels of support (moderate, strong, very strong running across the top) to the decision of whether a specific VUS is benign or pathogenic. Each individual supporting criteria has its own shorthand label in keeping with its position in the table. ‘Absent in population databases’ is PM2, standing for Pathogenic, Medium 2, while a variant in a ‘mutational hot spot or well-studied functional domain without benign variation’ is Pathogenic Medium, or PM1.

**Table 1.** 

	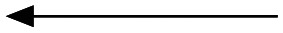 **Benign** 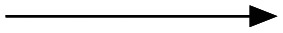		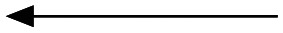 **Pathogenic** 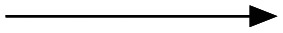			
	Strong	Supporting	Supporting	Moderate	Strong	Very strong
Population data	MAF is too high for disorder BA1/BS1 OR observation in controls inconsistent with disease penetrance BS2			Absent in population databases PM2	Prevalence in affecteds statistically increased over controls PS4	
Computational and predictive data		Multiple lines of computational evidence suggest no impact on gene /gene product BP4 Missense in gene where only truncating cause disease BP1 Silent variant with non-predicted splice impact BP7 In-frame indels in repeat w/out known function BP3	Multiple lines of computational evidence support a deleterious effect on the gene /gene product PP3	Novel missense change at an amino acid residue where a different pathogenic missense change has been seen before PM5 Protein length changing variant PM4	Same amino acid change as an established pathogenic variant PS1	Predicted null variant in a gene where LOF is a known mechanism of disease PVS1
Functional data	Well-established functional studies show no deleterious effect BS3		Missense in gene with low rate of benign missense variants and path. Missenses common PP2	Mutational hot spot or well-studied functional domain without benign variation PM1	Well-established functional studies show a deleterious effect PS3	
Segregation data	Nonsegregation with disease BS4		Co-segregation with disease in multiple affected family members PP1	**Increased segregation data** 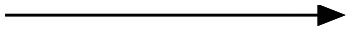		
De Novo data				De novo (without paternity & maternity confirmed) PM6	De novo (paternity and maternity confirmed) PS2	
Allelic data		Observed in trans with a dominant variant BP2 Observed in cis with a pathogenic variant BP2		For recessive disorders, detected in trans with a pathogenic variant PM3		
Other database		Reputable source w/out shared data = benign BP6	Reputable source = pathogenic PP5			
Other data		Found in case with an alternate cause BP5	Patient’s phenotype or FH highly specific for gene PP4			

In response to the question ‘shall I fire up your slide?’, Samantha, a colleague from the lab, replies:

Yeah. So, we found this *RPSka3* variant which is absent from the normal population, is highly conserved, and the computer tool seems to suggest that it's pathogenic and also the z-score, the ExAC constraint score, is positive. So you've got, from the ACMG guidelines: PM2, PP3, PP2 – you've got a VUS.

Unpacking this is useful for understanding the flavour of the discussion: The fact that the gene is highly conserved means that it is stable over evolutionary time, suggesting an important biological function and hence that variations may be pathogenic. A high Z score for constraint indicates a lower rate of benign variation, leading to fewer benign missense variants in a gene than expected. Thus, if the variant in question is a missense variant – a common mechanism of disease – it is more likely to be pathogenic. Exome Aggregation Consortium (ExAC) is a coalition of investigators seeking to aggregate and harmonize exome sequencing data from a wide variety of large-scale sequencing projects. PM2 means that the variant is absent in population databases, PP3 indicates that computational evidence supports deleterious effect, and PP2 describes a missense variant in a gene with low rate of benign missense variation.

In an attempt to move beyond this to a more certain result, Samantha then discusses another possible criterion: ‘But if you go onto the next slide, I thought maybe you could apply PM1 [located in a mutational hot spot] which would give it a “likely pathogenic” classification.’ Pointing at the slide, she suggests that the variant in question is in a mutational hotspot – a point on the genome with high mutation frequency – ‘because if those three variants at the top there are the only variants in that exon and yet all those pathogenic changes are in HGMD [Human Gene Mutation Database] in the same exon.’ As a result, that point on the genome can be characterized in line with the criteria for PM1:

Alan: So, it's a well characterized functional domain, in this case it's a protein kinase catalytic domain, with almost no …

Samantha: with no benign variation …

Alan: with no benign variation and a whole chunk of pathogenic mutations. So that would give us another moderate which would take us to two moderate and two supporting which would make it likely pathogenic …

Drawing on the ACMG standards, which set out how to ‘add up’ the various criteria to decide how to classify a variant, Alan and Samantha conclude that, with two moderate – in this case PM2 and PM1 – and two supporting criteria (PP3 and PP2), one can classify this variant as ‘likely pathogenic’ (or a ‘4’).

This relatively straightforward example highlights the role of various external resources (such as databases, computer-based tools to calculate pathogenicity) as well as more local interpretative practices (over whether the variant is in a mutational hot spot or not).

## Perceived variation in practice across the NHS and the challenges to standardization

What quickly became clear when we started our observations is that the nature of NHS genomic services^4^ – distributed between more than twenty regional centres – can act *against* reflexive standardization, by undermining trust in the decisions made at those other centres. The clinical exome service studied by Timmermans was largely independent of other testing centres; while applications might be received by clinicians from other parts of the country, testing and interpretation were largely carried out in-house. The interconnected nature of NHS clinical genomics, however, means that patients might arrive at the service with testing already run elsewhere in the country (certain centres specialize in specific conditions) and, perhaps, with familial test results from another centre.

Having acknowledged that variation in classificatory practice remains the case between NHS centres, despite the application of a shared standard, the obvious question is to explore those processes – such as differences between databases – that drive such variation. The following sections map out how knowledge of other centres in the NHS shapes VUS decision-making at Ernshire clinical genomics service, focusing on perceptions of other centres’ choices about technical aspects of sequencing (such as the kinds of test used, or access to relevant data) and variation in the interpretation of sequence data.

Among the professionals at Ernshire, differences between testing centres are seen, in part, to rest on variation in the technical choices involved in both sequencing of DNA (for example the choice of gene panels and the range and number of different variants sequenced at any one time) and the informatic resources drawn on to make sense of the variants that result. For example, in a case review meeting, the team discuss the result from another centre, where DNA from a patient suspected of having Hereditary Spastic Paraplegia (HSP) was put into an HSP panel, coming back with two variants associated with autosomal recessive spastic ataxia of Charlevoix-Saguenay (ARSACS), a rare condition normally only occurring in people from a specific region of Canada. Given the poor fit between this condition and the patient’s phenotype, discussion then moves onto whether the original choice of the HSP panel was the right one – or should it have gone to a neuropathy panel in the first instance? Andrea (consultant clinical geneticist) asks about the inclusion of the ARSACS gene on the HSP panel – it has led to ambiguous results before. Graham (consultant clinical geneticist) points out that there are different practices between different labs: ‘Some laboratories have really quite tight panels which fit the phenotype much more closely and other laboratories have panels where they’ll include every single gene that’s ever been associated with one of the symptoms.’

While, as set out below, much of the commentary on other centres’ classificatory practices can be seen as critical, there was also considerable awareness of how such variation could come about by quite legitimate means. As suggested by the previous scholarship, external resources, such as databases, computer programmes (to calculate pathogenicity for example) and journal articles play an important role in resolving a variant’s VUS Status. At the same time, of course, in terms of reflexive standardization, these resources are also key points, where variation in practice (and thus the need to trust other professionals’ decisions) becomes explicit.

This is clear in the scenario presented by Bill (consultant clinical geneticist) as an explicitly ‘cautionary tale’ of a premature baby with suspected cardiac problems (Long QT syndrome). DNA was sent to the genetics centre at Minton for testing, resulting in an ambiguously worded letter (‘their standard, “likely pathogenic” wording, but actually says we would advise testing in more, in more affected relatives first’) which Bill ‘would have read this as a class three [VUS] report actually, and so apparently did everybody else’.

While the original test was before Bill was in his post, he subsequently sent the material to another centre – Eveshalt – for their long QT panel:

because there are 16 genes [on the panel] now. As the result, the only thing that comes up is this variant [i.e. the same one as before] and Eveshalt say: ‘We now class this as a class four [i.e. ‘likely pathogenic’] in the light of new information.’ I say ‘Thank you, I will just run it past Minton as theirs is the original report.’ They email, say ‘Yes, still class four, but I'll just check it against ACMG.’ And it comes back as a class two [i.e. ‘likely benign’].

The explanation Bill offers for this significant variation in classification between centres lies in the upgrading of the resources available over time, specifically the evolution of one database (the Exome Aggregation Consortium or ExAC) into its successor (the Genome Aggregation Database or gnomAD). The key difference between the databases lies in size and the number of samples, with ExAC’s exome sequence data from 60,706 individuals being outpunched by gnomAD’s collection of 125,748 exomes and 15,708 genomes (Bahcall, 2016; Karczewski et al., 2017, 2020). In this specific case, Bill explains it in terms of:

What the difference is, people know the new gnomAD information … there is information in there that has not been let into ExAC so, although this is a coding variant, it was filtered out of ExAC, so it appeared that there was no population frequency.

While this variant ‘was not considered good enough data to get into ExAC, … it’s possible to see in gnomAD’.

Given that the time period over which we collected our data roughly corresponded to the increased use of gnomAD (and subsequent relegation of ExAC to the background), differences over the use of these databases are, perhaps, to be expected. Yet the move from ExAC to gnomAD is not without its drawbacks. While attempting to resolve a VUS in a panel testing for aortopathy in a Marfan’s patient, Alan points out that:

So we’d use PP3 in this case – which is that it’s a missense variant in a gene with low rate of mutation – based on ExAC constraint. [However], obviously the 2019 ACGS [Association for Clinical Genomic Science guidance] whatever criteria says we should be using gnomAD Z score instead. For some reason the increased data [in gnomAD] has reduced the Z scores, so actually many things which were above 3 are no longer above 3 if you go from ExAC to gnomAD, and this is an example of it, you would actually, if you reclassify using gnomAD, you'll lose that criteria so this one was a Z score over 3, but if you go to gnomAD, it's now 1.4. The new data in gnomAD is actually dropping …. I've actually found consistently on two or three genes, and actually it often tips things which used to be LP [likely pathogenic] into VUS.

Alan’s point is that upgrading the database one uses, from ExAC to gnomAD, can have the effect of making it harder to report out a VUS as pathogenic. These issues around external databases challenge the clinical genetics professionals at Ernshire to make decisions about, not just how other people apply the AMCG standards (i.e. reflexive standardization) but also the basis for the information in those databases and how they are constructed.

These kinds of choices are also reflected in the decisions these professionals have to make about the published literature. In a context where there may only be one or two papers linking a specific gene to a phenotype (and even then the variant may not be exactly the same as the one under investigation), clinicians are regularly faced with questions about the quality of the published literature.

Take, for example, a prenatal case: Ultrasound scanning of a foetus indicated that there were some unusual aspects to some of the bones, suggesting a case of skeletal dysplasia. An amniocentesis suggests a gain of genetic material in an area of a gene called *SHOX*, variants of which are associated with skeletal disorders. While the area of the gene in question is classified as benign,

we had another look at it and went into the literature. there seems to be one author [name] he’s actually done publications in 2011 and 2017 where he implies that SHOX duplications are implicated in skeletal dysplasias and complete SHOX dups have been reported in Leri-Weill.^5^ (Sally, laboratory colleague)

These papers link the phenotype under investigation with relevant kinds of variants in the gene in question, yet for Sally there are reasons to be cautious about taking these publications at face value since

most of what’s in the literature seems to come from this one [research group] and that worries me a little bit when it comes from one particular group so I think that those are the kinds of conversations we, I suppose, we need to decide how strong is the evidence and whether if it’s just from one place, what weight we want to put on that kind of evidence.

This concern crops up again later in the day when an ad hoc meeting is called to discuss this specific case,^6^ and Sally again points out the

need to discuss in a little bit more detail and I think he’s the only publication that seems to come out and sort of says this duplication is associated with short stature blah de blah de blah and might, and I think we need to keep, I suppose, an eye on it seems to be very limited evidence out there and it comes particularly from one author … there seems very little … keep coming back to this particular author and this particular group he seems to have quite a lot to say on the matter but from no-one else really.

Yet even in those cases where the team has confidence in the reliability of a paper, there remain questions of whether insights offered in a specific publication can be applied to the case under investigation. As Timmermans (2015, p. 87) notes, a key question for these meetings is ‘has this variant been seen before?’ Yet sometimes this is harder to answer than one might think, most obviously when one is trying to work out whether a partial duplication of a gene is the same, in clinical terms, as a whole gene duplication. The most obvious case of this in our data was discussed in a series of meetings over a 14-month period, with the problem being set out by Bob (consultant clinical geneticist) in a ‘Cases’ meeting at the genomics institute:

So it’s a partial duplication. So *ATRX*, as most of us will know, is a very important gene, and mutations within the gene and deletions within the gene, are related with X-linked learning difficulties, quite significant, in boys …

The puzzle to be solved is made clear in the ad hoc meeting from a couple of days earlier, arranged to deal with this specific case. Sally notes that the lab

could only find two publications…. So there’s one paper – 2007 – two sibs [siblings] with dups [duplications], but intragenic dups, of [exons] 2 to 35 and 2 to 29, so virtually the whole of the gene, so they’re not partial dups at all and there’s another paper in 2009 where they do report a partial dup but this time it does involve almost all the gene, from 2 to 31. But even with a partial dup of 2 to 31, they said there was some level of expression of the gene. So we’ve got nothing from the literature except possibly that particular case but it’s not identical.

The problem is that the duplications in the literature are much bigger than the comparatively small partial duplication in the case under consideration. And while these larger duplications may still mean the gene produces its protein, it is not clear that the same can be said of this smaller, partial dup. Sally spells this out:

[A]nd that’s the thing, this is such a difficult mechanism to try and work out because we really don’t know – in a partial dup when you’ve got a hit then in the gene, it’s really hard to know, because we know a whole duplication is nothing, but we don’t know if you have a part of it being affected, whether that is, as you say, disrupting the gene to cause an effect. We’re only hitting the last bit of it so you could say you might have some protein function up to the point where; is that enough to disrupt the whole gene?

Bob replies ‘I don’t know.’ With regard to the cases in the literature, when is a partial duplication not a partial duplication – perhaps when it is almost the whole gene.

In terms of reflexive standardization, questions about publications in the literature operate not just at the ‘second order’ level – Was the team who wrote this reliable? Are the duplications described here similar enough to our case to be relevant? – but speak directly to the ability of other genomics centres in the NHS to apply the ACMG standards properly: How other clinical geneticists read the literature tells one something about how reliably they apply the ACMG standards. For example, results come back on a panel test for a patient with potential Charcot-Marie-Tooth (CMT phenotype). A variant in the *HSPB1* gene is identified by the lab as a VUS. At the same time, the patient's son has been tested at another centre; there is some confusion over the dates (who was tested first) and the decision of a non-geneticist colleague to argue that because both have the variant it should be regarded as likely pathogenic.

In an effort to resolve the uncertainty, Graham went to the literature and found three articles on this variant that seemed to match the patient's phenotype; on closer inspection: ‘All three articles have the same mutation that’s been reported in this family but the original report [i.e. from the other centre] only quotes one article and it’s none of these three.’ While one of these three articles was published in 2018 (and thus, being recent, might be an acceptable oversight) the other two are from 2008 and 2011 and thus should have been discussed in the report from the other centre. This provokes Jane (genetic counsellor) into noting that ‘It seems with VUS now you end up doing, well, almost doing the interpretation again half the time.’ Graham notes that previous results from the lab in question have been reported as VUS but again, upon closer inspection, it is clear that there is more evidence available to resolve the result. This is couched in terms of effort, or lack of it on the part of the other lab: ‘It’s easier to report as a variant [of uncertain significance] without going off and doing this much work.’

## Variations in interpretation

Moving beyond technical variations – the types of tests applied, databases used and publications referred to – the professionals we studied also claimed insight into the interpretative practices of other centres and, over time, were able to build a perception of their more generalised standards. While it is a truism that any classification of a variant (as a VUS, benign or pathogenic) is temporary and can change over time as more information becomes available, also clear is that the ACMG criteria themselves serve as a driver of changing classifications, and of the perceived variation in practice between centres. Graham (consultant clinical geneticist) introduces discussion of a patient with a pituitary tumour causing blindness. Because the patient's aunt also had a pituitary tumour, material was sent to the lab at Holding for a panel, resulting in a variant in AIP (a gene associated with the inherited susceptibility to pituitary adenomas) that Holding claimed was pathogenic. However, investigating this variant on different databases resulted in four different classifications: ‘So it came up as being classified as benign, as a VUS, as being likely pathogenic and as being clearly pathogenic.’ There appear to be a number of reasons for this variation. In part, it is because there is very little published on it. There is a single 2010 paper published by the team at Holding, to which everyone refers, a kind of publication 'founder effect'; there is also another paper (which calls the variant in question a VUS) and some functional studies from Illumina, the company that makes the most common type of sequencing equipment used in the NHS. The result is that the report from the Holding lab is based on a classification that pre-dates the ACMG, with one of the clinical geneticists raising questions about Holding 's practice: ‘What will the [Holding] lab do when they get a variant which they know and they’ve probably got a list of variants, and they just classify it as pathogenic don't they, they don't go through and classify them all.’

In this case, the ACMG criteria become the driver of perceived variation between different centres – Holding’s failure to update its practice in line with the criteria undermine our participants’ confidence in these professionals’ application of standards. In a context where reflexive standardization is being applied, the introduction of a new, overarching external standard can actively undermine professionals’ trust in others’ ability to apply standards appropriately. The introduction of an overarching standard presents new opportunities for failure.

That other people are not making decisions in ways the Ernshire professionals view as correct becomes especially clear where these professionals are interpreting tests conducted at another centre. For example, in one meeting Rebecca (consultant clinical geneticist) articulates her discomfort at a case where members of the same family with DCM – dilated cardiomyopathy, a form of heart disease – have been tested at two other centres – Stanhurst and Minton – and have been found to share the same variant. The discussion at Ernshire focuses on what testing to offer a relative of these patients who has come to the clinic. The Stanhurst lab classified the variant as a VUS and thus Rebecca does not ‘feel comfortable to therefore offer a predictive test for this lady’. However, contradicting this is a senior clinician at Stanhurst, who ‘basically said it’s a truncating variant^7^ and it’s probably the cause of the cardiomyopathy’. Rebecca points out the dilemma: ‘It’s very difficult [to not offer testing] when you’ve got … somebody who’s an expert in another centre who’s saying that he would, he’s specifically written “I would offer cascade testing for family members.”’ As part of this discussion, reflecting on the different centres involved in testing this family, Graham notes that:

You’ll get a difference … the Minton lab would like to have absolutely every t crossed and i dotted before they will classify something as a 4 or a 5 [i.e. ‘likely pathogenic’ or ‘pathogenic’] whereas Stanhurst are almost the opposite. I’ve phoned them up and spoke to them before and they’ve said ‘As the clinician, if you’re happy to use it for pre-symptomatic testing we’ll call it a 4 [i.e. ‘likely pathogenic’].’

Similar concerns arise when the laboratories at other centres contradict what the Ernshire lab regards as good interpretative practice, as is the case in the so-called ‘slight mini-rant’ offered by the genetic counsellor Mia who has, on two recent occasions, had to give out results from other labs that the Ernshire lab wouldn't normally report. The first case concerns a pregnant woman whose nephew has a number of syndromic features (learning difficulties, a microcephaly) and who has been identified (by another lab) as having had an X chromosome deletion. The woman wanted a prenatal test for the deletion, but the Ernshire lab had a ‘look at it and they said, “well, there’s no genes the region at all, there’s nothing really there” and they said we wouldn’t have really reported that’. The second case involves a child with congenital heart defects whose array discovered a small deletion within the *DMD* gene which, when checked by the Ernshire lab: ‘they said, ”well it’s, it’s intronic, it’s deep intronic, it doesn't effect any boundaries. Again, we wouldn't have reported it.” ’ For Mia, best practice in interpreting these results, and thus in also applying the ACMG standards, can be found at the Ernshire lab. Yet these results from other test centres have to be reported to patients and their families, even if Ernshire will not act on them (by offering familial testing, for example).

While the genetics professionals we observed tended to prioritize the interpretations and testing practices of their own centre, a more nuanced view became clear over the course of our observations. The distributed nature of expertise across the network of genomics centres in the UK means that sometimes other centres have access to more detailed information – perhaps as the result of a local research project – and interpretative experience, with some centres specializing in testing for specific conditions. Awareness of this kind of ‘private’ information shapes how the Ernshire genetics professionals view the application of the ACMG standards by some other centres. For example, in a case of a family with a history of various cancers, Mary (consultant clinical geneticist) discusses a variant in the *FH* gene (associated with hereditary leiomyomatosis and renal cell cancer, HLRCC), and how:

In Ireland they were very much treating that *FH* variant as ‘likely pathogenic’ and they were testing unaffected people, which we were very uncomfortable about, because it just didn't really seem to be clear why. … And on the [Monday] morning … I opened my post to find an updated variant report … that had been sent to me from Ireland, but done in Hillwood to say that this was now reclassified as ‘likely pathogenic’, so theoretically, predictive testing could be offered.

Mary’s concerns were consolidated when she asked a member of the Ernshire lab to look at the variant and apply the ACMG criteria; it came back as a VUS.

So that was a bit weird, so I rang the [Hillwood] lab, thought it would be quite helpful to say to them why did they reclassify it?

And I spoke to the scientist in Hillwood, the Hillwood lab, and she was very helpful actually as she said it was to do with the segregation and the level of segregation that they have, which I think is stuff that we didn’t have and I think they’ve got more phenotypic data than we’ve got. So she said there are about four or five meiosis in that family that have been informative, so I think that’s why they’ve reclassified it so I think I’m reassured that we can offer them [i.e. the family] predictive testing for the *FH* variant, which is what I will do.

In this instance, the discrepancy between Ernshire and another centre’s classification is resolved easily, perhaps because there is a strong perception on the part of the professionals we observed that choosing to classify a variant in contradiction to the decisions made elsewhere – scoring what another centre calls a VUS as ‘likely pathogenic’ for example – has knock on effects. The choices made by Ernshire’s clinical geneticists do not take place in isolation but as part of a system, and hence the concerns mapped out towards the end of a discussion about a variant in the *GPD1* gene, which causes transient infantile hypertriglyceridemia. While the details of this case can be set to one side, the discussion has centred on two other patients with this variant – one in Hillwood, and the other in Adam Vale – who could be used as a resource to help classify the variant in question as ‘likely pathogenic’. However, at the two other centres the variant in question has already been classified as a VUS, raising a dilemma for the Ernshire professionals:

James (registrar clinical geneticist): The thing is if we, and I know that sometimes it has to be, somebody has to go forward and make that leap towards making that decision once we do that presumably their variant will be potentially re-classified.

Alan (consultant clinical geneticist): Potentially 'cause you classified it like yeah, you have a conflict for classification, potentially yeah.

Bill (consultant clinical geneticist): You all have to jump together.

Jane (genetic counsellor): Yeah, and they're saying they're not jumping. Think about it – In the list of for or against like how does that add up? Not ACMG for and against, our own common sense for and against.

In a system where individual centres’ classificatory decisions are interlinked, where choices made at Ernshire mean that at other centres a ‘variant will be potentially re-classified’, it is important to coordinate decisions, ‘to all jump together’.

## Discussion

With an analysis based on 290 separate clinical meetings, attended over a two-year period, this article presents an ethnographic account of decision-making around NGS technology in a NHS clinical genomics service, broadening our understanding of the role formal criteria play in the classification of VUS. Building on previous studies that have concentrated on the use of specific, new NGS technologies – for example clinical exome sequencing (Skinner et al., 2016; Timmermans, 2015, 2017) or Whole Genome Sequencing (Sanderson et al., 2019) – this article explores a context in which a series of different genomic technologies might be applied, perhaps along a pathway, highlighting the integrated nature of such technologies in NHS testing. The results of a specific test are simply one of a number of different technologies used in the NHS context, into which NGS techniques are being introduced in parallel with other tests.^8^ In the NHS context, the discussion and decision making around VUS arising out of exome sequencing, or indeed WGS, are not qualitatively different – nor do they draw on different informational support – than discussions of VUS arising out of other kinds of test.

The first insight from our examination of VUS decision making – that the application of standards in this context is flexible and locally contingent – is, from an STS-perspective, obvious, and even banal. As already noted, work on the sociology of standards (Timmermans & Epstein, 2010), including considerable contributions from within STS (e.g. Abraham, 1993; Castel, 2009; Halverson, 2019) has emphasized how flexible standards come to be when interpreted on the ground, serving as a powerful resource for different interest groups.

More noteworthy are the kinds of variations mapped out in our data, especially given that the central aims behind the introduction of the ACMG criteria into NHS clinical genetics practice are to reduce variation in interpretation between testing centres and to standardize how VUS, for example, get dealt with. Yet while the standards set out in the ACMG criteria are more formalized and externally drafted than in Timmerman’s case, clearly his concept of reflexive standardization – the need to trust other professionals in their application of standards – remains acutely relevant. Even with strict guidelines like the ACMG criteria, panels still need to make decisions about trusting other people’s application of the criteria.

The standardized context within which these decisions are made – that is, the NHS genomic medicine service – makes reflexive standardization *harder*, since it gives clearer insight into, and increased scepticism about, variation in practices taking place across the system. While the obvious assumption would be that the US system examined by Timmermans would present more barriers to reflexive standardization than the more homogenous environment presented by the UK NHS, in our data the need for reflexive standardization in the application of ACMG criteria is not *in spite* of the NHS and its genomic medicine service but because of them. The organization of genomic sequencing within the NHS, with connections between centres giving insight into other professionals’ practice, provides a setting in which trust in others’ application of standards (or lack of it) remains central to decision making. Whether it is the perceived differences between Minton and Stanhurst over willingness to class a variant as ‘likely pathogenic’, variation over how tightly certain gene panels are seen to fit phenotypes, or some centres’ perceived lack of effort around variant interpretation, Ernshire’s clinical geneticists and their colleagues judge the ability and practice of other centres elsewhere in the system. These judgments, in turn, feed into their own decisions about pathogenicity and uncertainty, in the same way as judgements about the reliability of the published literature and genome databases (for a prescient discussion see Kim et al., 2019).

One solution to such variation would be further coordination and standardization, to turn the handle one more time and ensure all genomic centres are applying the same rules in the same way. Such a perspective is in keeping with the original discussions around the need to adopt the ACMG criteria:

The ability of all NHS genetics laboratories to reach a consistent and accurate interpretation of a particular variant is paramount given the implications for patient management and safety. Key to driving this consistency is the use of a common set of principles and approach to assessing variants (Raza et al., 2017, p.5).

Indeed, there is professional concern about consistency within the UK in the reporting of genetic variants and especially the reclassification of variants, although the costs of setting up mechanisms to achieve this at scale would be substantial. In the context of cancer, there are proposals for a national review of reclassifications that downgrade the disease risk associated with a variant if it would lead to altered clinical practice (e.g. downgrading the intensity of disease surveillance) (Loong et al., 2022). More generally, the professional organizations involved have called for mechanisms to ensure consistency in variant interpretation and reinterpretation across the NHS (Ellard et al., 2020, Section 7, pp. 29–30).

As noted in the discussion above of the *GPD1* gene, the professionals we observed are acutely aware that the classificatory choices they make will be communicated to other centres in the service. This interconnection – how information on one centre’s classification decisions is shared with other centres – complicates the nature of the reflexive standardization taking place. While the application of standards at another centre shapes Ernshire’ own application of standards (i.e. reflexive standardization), that in turn – through letters, publications and entries in databases – shapes later application of standards at those other centres.

And professionals’ awareness of this feedback mechanism, in turn, shapes decisions at the Ernshire centre. The clearest example of this ‘*re*-reflexive standardization’ arises out of the knowledge that classing a variant as ‘pathogenic’ or ‘likely pathogenic’ will make it eligible for use in prenatal testing, opening up the possibility that a termination of pregnancy may take place based on that classification. On a number of occasions we observed this possibility – that other professionals could make such a decision based on Ernshire’s classification – feeding back into the original decision and persuading professionals to leave a variant as a VUS.

In essence, if the core question of straightforward standardization is ‘how do we apply these standards’, and of reflexive standardization is ‘how does how they apply these standards effect how we apply these standards’ then the core question of re-reflexive standardization is ‘how does how they apply these standards effect how we apply these standards and how, in turn, does this effect how they will apply these standards in the future?’

Taking this insight forward into sociological studies of genomics would require analysis to include consideration of the broader system within which individual clinics or testing centres operate, moving beyond claims that isolate the group making decisions about genomic diagnoses and placing them in a broader context.

## Notes

1. The patient-facing element of our research was also extensive, involving observation and recording of 30 clinical consultations where the results of genetic/genomic tests were fed back to patients/family members. However, this current paper focuses on the internal professional discussions and thus does not draw on this dataset.

2. ACMG criteria are referred to in the Belgium testing centre explored by Kuiper et al. (2022), but they are presented as marginal to the decision-making process, mentioned ‘often in passing and without much specificity’ (p. 7).

3. This description draws on the classic appeal to visual cues adopted by dysmorphology, a longstanding area of specialism within clinical genetics and a topic of considerable interest on the part of sociologists in this area (Latimer, 2013; Latimer et al., 2006).

4. A note on terminology: while current policy discussions emphasize the term clinical genomics – for example, the NHS now has a ‘Genomic Medicine Service’ – the professionals we observed talk about themselves in terms of ‘genetics’, as clinical geneticists or genetic counsellors. While ‘genomics’ is used to refer to specific technologies, it tends not to be referred to in terms of professional identity. Here we try to map onto our participants’ language use, talking about them as geneticists, who interpret genetic and genomic tests, in a clinical service which has recently been rebranded in terms of a ‘medical genomics service’.

5. Leri-Weill dyschondrosteosis (LWD) is a rare genetic disorder characterized by, amongst other things, abnormal shortening of the forearms and lower legs, and short stature.

6. Ad hoc meetings are arranged when complex VUS are brought to the weekly prenatal team meeting. The prenatal meetings themselves are brisk and business like, the main aim being to update the team on where specific patients are on the list and whether they have moved off. These characteristics are driven by the singular intervention available to patient on this list – termination of pregnancy – which is itself extremely time-limited. Thus complex cases like this one are not discussed in detail at prenatal meetings but rather are deferred to an ad hoc meeting that takes place usually later the same day.

7. A truncating variant is a genetic variant which results in a shorter than normal version of the protein being produced.

8. Not least because, as already pointed out, in the NHS most WGS takes the form of ‘virtual panels’, where interpretation is focused on a number of areas associated with the phenotype in question.
